# Diffusion MRI-based cortical connectome reconstruction: dependency on tractography procedures and neuroanatomical characteristics

**DOI:** 10.1007/s00429-018-1628-y

**Published:** 2018-02-20

**Authors:** Michel R. T. Sinke, Willem M. Otte, Daan Christiaens, Oliver Schmitt, Alexander Leemans, Annette van der Toorn, R. Angela Sarabdjitsingh, Marian Joëls, Rick M. Dijkhuizen

**Affiliations:** 1 Biomedical MR Imaging and Spectroscopy Group, Center for Image Sciences, University Medical Center Utrecht/Utrecht University, Yalelaan 2, 3584 CM Utrecht, The Netherlands; 2 Department of Pediatric Neurology, Brain Center Rudolf Magnus, University Medical Center Utrecht/Utrecht University, Utrecht, The Netherlands; 3 Department of Electrical Engineering, KU Leuven, ESAT/PSI, Leuven, Belgium; 4 Division of Imaging Sciences and Biomedical Engineering, King’s College London, London, UK; 5 Department of Anatomy, University of Rostock, Rostock, Germany; 6 Image Sciences Institute, University Medical Center Utrecht/Utrecht University, Utrecht, The Netherlands; 7 Department of Translational Neuroscience, Brain Center Rudolf Magnus, University Medical Center Utrecht/Utrecht University, Utrecht, The Netherlands; 8 University Medical Center Groningen, University of Groningen, Groningen, The Netherlands

**Keywords:** Brain, Brain connectomics, Diffusion MRI, Diffusion tractography, Constrained spherical deconvolution, Neuronal tracers, Rats

## Abstract

Diffusion MRI (dMRI)-based tractography offers unique abilities to map whole-brain structural connections in human and animal brains. However, dMRI-based tractography indirectly measures white matter tracts, with suboptimal accuracy and reliability. Recently, sophisticated methods including constrained spherical deconvolution (CSD) and global tractography have been developed to improve tract reconstructions through modeling of more complex fiber orientations. Our study aimed to determine the accuracy of connectome reconstruction for three dMRI-based tractography approaches: diffusion tensor (DT)-based, CSD-based and global tractography. Therefore, we validated whole brain structural connectome reconstructions based on ten ultrahigh-resolution dMRI rat brain scans and 106 cortical regions, from which varying tractography parameters were compared against standardized neuronal tracer data. All tested tractography methods generated considerable numbers of false positive and false negative connections. There was a parameter range trade-off between sensitivity: 0.06–0.63 interhemispherically and 0.22–0.86 intrahemispherically; and specificity: 0.99–0.60 interhemispherically and 0.99–0.23 intrahemispherically. Furthermore, performance of all tractography methods decreased with increasing spatial distance between connected regions. Similar patterns and trade-offs were found, when we applied spherical deconvolution informed filtering of tractograms, streamline thresholding and group-based average network thresholding. Despite the potential of CSD-based and global tractography to handle complex fiber orientations at voxel level, reconstruction accuracy, especially for long-distance connections, remains a challenge. Hence, connectome reconstruction benefits from varying parameter settings and combination of tractography methods to account for anatomical variation of neuronal pathways.

**Electronic supplementary material:**

The online version of this article (10.1007/s00429-018-1628-y) contains supplementary material, which is available to authorized users.

## Introduction

Mapping brain-wide structural connectivity has become an important goal in neuroscience and is the primary focus of the field of connectomics (Sporns et al. 2005; Sporns 2010). This field aims to construct comprehensive maps of all neuronal elements and their connections in different organisms, including a wide range of mammalian species. Connectomes can provide crucial insights into brain functioning in health and disease as (changes in) structural connections underlie at least part of functional and behavioral phenotypes (Sporns et al. 2005; Bassett and Bullmore 2009; Sporns 2010; Stam 2014).

Unraveling the human connectome is a great challenge (Poldrack and Farah 2015) and its reconstruction would be a fundamental breakthrough in neuroscience (Sporns et al. 2005; Sporns 2010). So far, a complete connectome has been mapped only for *C. elegans* (White et al. 1986). Accurate connectome reconstructions at micro-, meso- and macro-scale in other species, including *Drosophila* (Chiang et al. 2011), mouse (Oh et al. 2014), rat (Schmitt and Eipert 2012), macaque (Stephan et al. 2001) and human (Van Essen et al. 2012) involve ongoing efforts in large-scale multicenter projects, such as the Human Connectome Project (Van Essen et al. 2012), the BRAIN project (NIH 2014), the Brainnetome Project (Jiang 2013) and the CONNECT project (Assaf et al. 2013).

Diffusion MRI-based tractography has offered exclusive means to map structural connections non-invasively in animal and human brain, and is considered a very useful and powerful technique to unravel the human connectome at meso- and macro-level (Le Bihan et al. 1986; Basser et al. 1994b; Mori et al. 1999; Jones 2008; Jbabdi and Johansen-Berg 2011). However, the anatomical accuracy of diffusion-weighted tractography, which infers neuronal pathways from tissue water diffusion direction, continues to be a matter of controversy, and reconstructing connectomes with both high sensitivity and high specificity remains challenging (Sporns et al. 2005; Bastiani et al. 2012; Jbabdi et al. 2013, 2015; Thomas et al. 2014; Azadbakht et al. 2015; Calabrese et al. 2015; Knösche et al. 2015; Reveley et al. 2015; Donahue et al. 2016). This may lead to biased characterization of connectome topology, particularly in the presence of false positive tract reconstructions (Drakesmith et al. 2015; Zalesky et al. 2016).

The classical approach of diffusion tensor (DT)-based deterministic tractography (Basser et al. 2000) uses the diffusion tensor model (i.e., estimating one principal direction in each voxel) to reconstruct fiber tracts in the brain. This method is particularly hampered by voxels containing multiple fiber orientations such as crossing, bending or fanning fibers (Jbabdi and Johansen-Berg 2011). At standard spatial resolutions around a few mm, multiple fiber orientations affect the diffusivity patterns in up to 90% of white matter voxels in the human brain (Jeurissen et al. 2013). To improve the characterization of the intravoxel diffusion profile and the accuracy of subsequent tractography, high-angular resolution diffusion imaging (HARDI) methods have been developed (e.g., Descoteaux et al. 2007; Jeurissen et al. 2009; Tournier et al. 2011; Tax et al. 2014). For instance, constrained spherical deconvolution (CSD), developed by Tournier et al., models the diffusion profile in voxels with multiple fiber orientations (Tournier et al. 2004, 2007). Instead of estimating one principal fiber direction, CSD estimates multiple fiber directions within a voxel, resulting in a fiber orientation distribution (FOD) that may have multiple peaks (e.g., four in case of two crossing fibers in one voxel) (Tournier et al. 2010, 2012). CSD-based tractography uses second-order integration over fiber orientation distributions, thereby including information from orientations in neighboring voxels (Tournier et al. 2010, 2012). This solves some of the typical tractography errors, but false positive tract reconstructions may still arise from noisy (spurious) peaks or from ambiguous fanning and bending fiber populations (Jbabdi and Johansen-Berg 2011). Recently, a new method, called spherical-deconvolution informed filtering of tractograms (SIFT) has been proposed to reduce local streamline density bias by filtering the tractogram (i.e., selectively removing streamlines) to improve the fit between the overall distribution of streamlines and the FOD in every voxel, estimated from the diffusion-weighted image, which may improve accuracy of connectome reconstruction (Smith et al. 2013, 2015a). Still, additional errors may arise from the conventional method of reconstructing tracts from streamlines that are propagated in a step-wise approach. Consequently, long-distance connections are more difficult to track, as the reconstruction error accumulates with each tracking step (Zalesky and Fornito 2009; Jbabdi et al. 2015; Reveley et al. 2015; Donahue et al. 2016). Instead of tracking streamlines, alternative tractography approaches model tracts at a global rather than voxel level, to solve local reconstruction issues with bending and fanning fiber populations as well as to mitigate the reconstruction error accumulation at longer distances. Global tractography aims to find the full track (i.e., whole brain) configuration that most optimally fits the acquired data (Reisert et al. 2011; Mangin et al. 2013; Christiaens et al. 2015b). Modern methods such as CSD-based and, global tractography and SIFT may significantly improve the performance of brain-wide tractography and contribute to more accurate connectome reconstructions. However, anatomical validation is largely lacking.

Validation studies in mammalian species from which neuronal tracing data is available, offer powerful means to directly compare diffusion-based tract reconstructions against true neuroanatomical connectivity (Dauguet et al. 2007; Dyrby et al. 2007; Gao et al. 2013; Thomas et al. 2014; Calabrese et al. 2015; Chen et al. 2015). This approach has been successfully applied to validate tractography methods and/or to verify reconstruction of specific white matter structures in squirrel monkeys (Gao et al. 2013), mini-pigs (Dyrby et al. 2007) and macaques (Thomas et al. 2014; Reveley et al. 2015). However, validation of whole-brain connectome reconstruction (i.e., structural connectivity between multiple brain regions) from contemporary diffusion-based tractography approaches is currently lacking. Therefore, we compared brain-wide structural connectome reconstruction from DT-based, CSD-based and global tractography, using ultrahigh-resolution diffusion-weighted images of rat brain, against neuroanatomical connectivity measures from tracer studies. We evaluated tractography results across different anatomical sites and distances in the brain and determined how parameter settings affect the connectome reconstruction. In addition, we assessed the effect of SIFT, streamline thresholding and group-based average network thresholding on connectome reconstructions.

## Methods

### Animal preparation

All animal procedures were approved by the Animal Experiments Committee of the University Medical Center Utrecht and Utrecht University, and experiments were performed in accordance with the guidelines of the European Communities Council Directive. Ten healthy adult (12–13 weeks old) male Wistar rats that were randomly distributed for an earlier study (Dijkhuizen et al. 2017) were group-housed under standard conditions with light/darks cycle on/off from 07:00 to 19:00. Animals were sacrificed in the morning between 09:00 and 12:00 by intraperitoneal injection of an overdose of pentobarbital, followed by transcardial perfusion-fixation with 4% paraformaldehyde in phosphate-buffered saline. Brains inside the intact skull were extracted and positioned in a container filled with proton-free oil (Fomblin®) for MRI scanning.

### Diffusion MRI

Diffusion-weighted imaging was performed with a 9.4 T small-animal MR scanner (Varian, Palo Alto, CA, USA) and a custom-made solenoid coil with an internal diameter of 2.6 cm. High spatial and angular resolution diffusion imaging (HARDI) was accomplished using an 8-shot 3D echo planar imaging (EPI) sequence, with repetition time (TR) = 500 ms, echo time (TE) = 32.4 ms, Δ/δ 15/4 ms and *b* value = 3842s/mm^2^. The field-of-view (FOV) was 19.2 × 16 × 33 mm^3^ and the data matrix size was 128 × 108 × 220, resulting in a voxel resolution of 150 × 148 × 150 µm^3^. The HARDI protocol consisted of acquisition of five non-diffusion-weighted (*b*_0_) images and 60 images with different non-collinear diffusion-weighting directions on a half sphere. Total acquisition time was 8 h per sample.

### Data processing

Preprocessing was done with FSL (https://www.fmrib.ox.ac.uk/fsl/) (Jenkinson et al. 2012) and included brain extraction (Smith 2002) and registration steps. Correction for eddy currents or bias field was not necessary, because we used a special volume coil and MRI pulse sequence (with extra delays after sinus-shaped gradients) that allowed homogenous imaging of post mortem brain tissue. With FLIRT (Jenkinson and Smith 2001; Jenkinson et al. 2002) and FNIRT (Andersson et al. 2007), diffusion-weighted images of individual rat brains were linearly and non-rigidly aligned to a rat brain template matched with a custom-built 3D model (Majka et al. 2012) of the 5th edition of the Paxinos and Watson rat brain atlas (Paxinos and Watson 2005). All 106 bilateral atlas regions that cover the entire cortex were subsequently back-projected into subject space for subsequent tractography.

### Diffusion-based tractography

All diffusion-based tractography approaches and subsequent connectome reconstructions were performed in MRtrix3® (https://www.mrtrix.org/) (Tournier et al. 2012).

#### DT-based tractography

The DT model was directly estimated from the raw diffusion-weighted data using standard log-linear least-squares regression (Basser et al. 1994a). Fractional anisotropy (FA) maps were determined from the diffusion tensor. Streamlines were generated from randomly placed seeds over a mask covering the whole brain. We varied the following parameters for streamline computation: step size (15, 30 and 50 µm), FA threshold (0.05, 0.10, 0.15 and 0.20) (streamlines will terminate in voxels with a sub-threshold FA value), angular threshold (20–80° with steps of 10°) (streamlines will terminate when bending with an angle exceeding the threshold), and number of streamlines (25, 50, 100 and 250 × 10^3^).

#### CSD-based tractography

The CSD-model was computed from the signal amplitude yielding FOD maps used for CSD-based tractography. Streamlines were generated from randomly placed seeds over a brain mask. We varied the following tractography parameters: step size (50, 75 and 100 µm), angular threshold (20–80° with steps of 10°), number of streamlines (25, 50, 100 and 250 × 10^3^), and FOD amplitude threshold (0.1, 0.125, 0.15, 0.175). Higher FOD amplitude thresholds will cause a more rigid delineation of tracts, since streamlines will not follow directions with low FOD amplitudes, whereas lower thresholds will lead to more variation in tract reconstructions.

#### Global tractography

Global tractography uses the estimation of distinct diffusion profiles (i.e., response functions) for different tissue classes (i.e., white matter, gray matter and cerebrospinal fluid) to deal with partial volume effects. Because the postmortem rat brains in our study contained relatively little cerebrospinal fluid and were embedded in proton-free oil, we restricted tissue classification to gray and white matter. In global tractography, tracts are built from segments, called particles (default length was 100 µm for our dataset). In line with a previous validation study in human brain (Christiaens et al. 2015b), we varied two key parameters, i.e., particle potential (0.1, 0.2, 0.5, 1, 2 and 5) and connection potential (0.01, 0.02, 0.05, 0.1, 0.2 and 0.5). The particle potential regulates the number and distribution of particles, where higher values weigh towards larger white matter structures (i.e., less particles in cortical areas), whereas lower values increase the number of particles in structures like the cortex. The connection potential drives the connection of these segments (i.e., particles) and thereby the formations of tracts, where higher values increase the formation of reconstructed tracts.

#### Spherical deconvolution informed filtering of tractograms (SIFT)

We applied SIFT (Smith et al. 2013, 2015a) on DT-based and CSD-based tractograms, which optimizes and fits the reconstructed white matter tracts (i.e., streamlines) to the underlying diffusion-weighted images. This approach normalizes and corrects the number of streamlines (i.e., filters the tractogram) based on spherical deconvolution of the diffusion signal. This leads to a data-driven decrease in network density, without requirement of an arbitrary threshold (such as a streamline threshold). We evaluated the effect of SIFT correction compared to standard CSD-based and DT-based tractography for 250,000 streamlines (since a high number of streamlines is required for SIFT). We limited our analysis to the default step sizes for DT- and CSD-based tractography, two FOD/FA thresholds and four angle thresholds, to manage computational time.

### Diffusion tractography-based connectome reconstruction

Connectomes were constructed by comparing, for each brain, generated tracks between 106 cortical regions from the Paxinos and Watson rat brain atlas (Table 1). Two regions were considered to be structurally connected if one or more streamlines had their endpoints in both regions. The number of streamlines connecting pairs of regions was ignored; i.e., all connections were binarized.

**Table 1:** For all included cortical regions (*N* = 106) the Paxinos and Watson atlas region of interest (ROI) label and full anatomical names are shown

Cortical regions used for connectome constructions					
Nr	ROI-label	Anatomical region	Nr	ROI-label	Anatomical region
1	AID	Agranular insular cortex dorsal part left	54	PtPC	Parietal cortex posterior area caudal part right
2	AID	Agranular insular cortex dorsal part right	55	PtPD	Parietal cortex posterior area dorsal part left
3	AIP	Agranular insular cortex posterior part left	56	PtPD	Parietal cortex posterior area dorsal part right
4	AIP	Agranular insular cortex posterior part right	57	PtPR	Parietal cortex posterior area rostral part left
5	AIV	Agranular insular cortex ventral part left	58	PtPR	Parietal cortex posterior area rostral part right
6	AIV	Agranular insular cortex ventral part right	59	RSD	Retrosplenial dorsal left
7	Au1	Primary auditory cortex left	60	RSD	Retrosplenial dorsal right
8	Au1	Primary auditory cortex right	61	RSGa	Retrosplenial granular cortex a region left
9	AuD	Secondary auditory cortex dorsal area left	62	RSGa	Retrosplenial granular cortex a region right
10	AuD	Secondary auditory cortex dorsal area right	63	RSGb	Retrosplenial granular cortex b region left
11	AuV	Secondary auditory cortex ventral area left	64	RSGb	Retrosplenial granular cortex b region right
12	AuV	Secondary auditory cortex ventral area right	65	RSGc	Retrosplenial granular cortex c region left
13	Cg1	Cingulate cortex area 1 left	66	RSGc	Retrosplenial granular cortex c region right
14	Cg1	Cingulate cortex area 1 right	67	S1	Primary somatosensory cortex left
15	Cg2	Cingulate cortex area 2 left	68	S1	Primary somatosensory cortex right
16	Cg2	Cingulate cortex area 2 right	69	S1BFa	Primary somatosensory cortex barrel field left
17	DI	Dysgranular insular cortex left	70	S1BFa	Primary somatosensory cortex barrel field right
18	DI	Dysgranular insular cortex right	71	S1DZ	Primary somatosensory cortex dysgranular region left
19	DIEnt	Dorsal intermediate entorhinal cortex	72	S1DZ	Primary somatosensory cortex dysgranular region right
20	DIEnt	Dorsal intermediate entorhinal cortex	73	S1DZO	Primary somatosensory cortex oral dysgranular region left
21	DLEnt	Dorsolateral entorhinal cortex left	74	S1DZO	Primary somatosensory cortex oral dysgranular region right
22	DLEnt	Dorsolateral entorhinal cortex right	75	S1FL	Primary somatosensory cortex forelimb region left
23	DLO	Dorsolateral orbital cortex left	76	S1FL	Primary somatosensory cortex forelimb region right
24	DLO	Dorsolateral orbital cortex right	77	S1HL	Primary somatosensory cortex hindlimb region left
25	Ect	Ectorhinal cortex left	78	S1HL	Primary somatosensory cortex hindlimb region right
26	Ect	Ectorhinal cortex right	79	S1J	Primary somatosensory cortex jaw region left
27	Fr3	Frontal cortex area 3 left	80	S1J	Primary somatosensory cortex jaw region right
28	Fr3	Frontal cortex area 3 right	81	S1Sh	Primary somatosensory cortex shoulder region left
29	FrA	Frontal association cortex left	82	S1Sh	Primary somatosensory cortex shoulder region right
30	FrA	Frontal association cortex right	83	S1Tr	Primary somatosensory cortex trunk region left
31	GI	Granular insular cortex left	84	S1Tr	Primary somatosensory cortex trunk region right
32	GI	Granular insular cortex right	85	S1ULp	Primary somatosensory cortex upper lip region left
33	IL	Infralimbic cortex left	86	S1ULp	Primary somatosensory cortex upper lip region right
34	IL	Infralimbic cortex right	87	S2	Secondary somatosensory cortex left
35	LEnt	Lateral entorhinal cortex left	88	S2	Secondary somatosensory cortex right
36	LEnt	Lateral entorhinal cortex right	89	TeA	Temporal association cortex 1 left
37	LO	Lateral orbital cortex left	90	TeA	Temporal association cortex 1 right
38	LO	Lateral orbital cortex right	91	V1	Primary visual cortex left
39	LPtA	Lateral parietal association cortex left	92	V1	Primary visual cortex right
40	LPtA	Lateral parietal association cortex right	93	V1B	Primary visual cortex binocular area left
41	M1	Lateral agranular prefrontal cortex left	94	V1B	Primary visual cortex binocular area right
42	M1	Lateral agranular prefrontal cortex right	95	V1M	Primary visual cortex monocular area left
43	M2	Medial agranular prefrontal cortex left	96	V1M	Primary visual cortex monocular area right
44	M2	Medial agranular prefrontal cortex right	97	V2L	Secondary visual cortex lateral area left
45	MO	Medial orbital cortex left	98	V2L	Secondary visual cortex lateral area right
46	MO	Medial orbital cortex right	99	V2ML	Secondary visual cortex medial area left
47	mPFC	Medial prefrontal cortex left	100	V2ML	Secondary visual cortex medial area right
48	mPFC	Medial prefrontal cortex right	101	V2MM	Secondary visual cortex mediomedial area left
49	MPtA	Medial parietal association cortex left	102	V2MM	Secondary visual cortex mediomedial area right
50	MPtA	Medial parietal association cortex right	103	VIEnt	Ventral intermediate entorhinal cortex left
51	PRh	Perirhinal cortex left	104	VIEnt	Ventral intermediate entorhinal cortex right
52	PRh	Perirhinal cortex right	105	VO	Ventral orbital cortex left
53	PtPC	Parietal cortex posterior area caudal part left	106	VO	Ventral orbital cortex right

### Neuroanatomical tracing database

Neuronal tracer data were extracted from the rat connectome database (https://neuroviisas.med.uni-rostock.de/connectome/index.php) that was built with neuroVIISAS (Schmitt and Eipert 2012). This database contains more than 450,237 ipsi- and 175,654 contralateral connections from more than 6183 peer reviewed publications which describe connectivity observations based on anterograde and retrograde neuronal tracer injections in normal juvenile or adult rats. This tracer-based connectome covers all cortical atlas regions. We extracted connectivity information for the same 106 cortical regions (Table 1) as used for diffusion-based connectome reconstructions. Several tracer-based connections (between pairs of regions) are unidirectional. We considered two regions neuroanatomically connected irrespective of directionality. As such, the tracer-based connectome had a network density (i.e., the proportion of true connections out of all potential connections) of 0.27 (Figure S1).

### Quality of diffusion tractography-based reconstructions

#### Sensitivity, specificity and Jaccard index

**Fig. 1:**
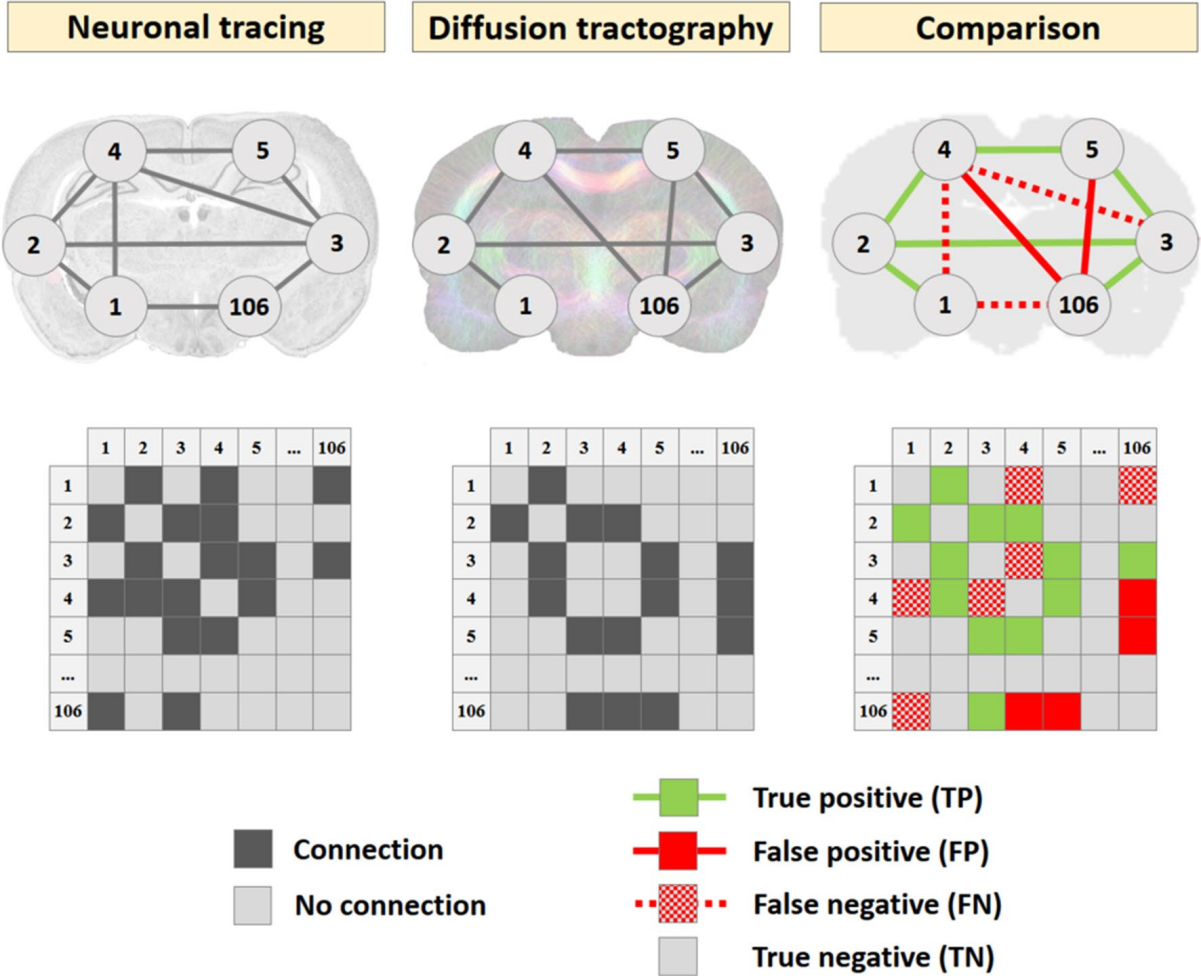
Comparison of connectivity networks from neuronal tracer database and diffusion tractography algorithms. Neuronal tracer-based (left column) and diffusion tractography-based (middle column) connectivity networks represented as network graphs (top), in which nodes represent cortical atlas regions (*N* = 106) and edges represent connections, and as adjacency matrix (bottom), in which rows and columns represent cortical regions and dark squares represent connections. Diffusion tractography-based connectivity networks were compared against the neuronal tracer-based network as ground truth, which yielded true positives (green lines and squares), false positives (red lines and squares), false negatives (dotted red lines and squares), and true negatives (no line and color-coding) (right column).

The diffusion tractography-based connectivity (i.e., structural connectome) was compared against neuronal tracer-based connectivity, as schematically illustrated in Fig. 1. Connectome reconstruction performance for the different tractography algorithms was evaluated with three different measures.

(1) Sensitivity, which is the proportion of correctly identified present connections (also known as true positive rate; TPR), i.e., the number of true positives (TP) divided by either the number of present connections (P) or the summation of true positives and false negatives (FN):

1 $${\text{Sensitivity}}\;\left( {{\text{TPR}}} \right)={\text{TP/}}P={\text{TP/}}\left( {{\text{TP}}+{\text{FN}}} \right).$$

(2) Specificity, which is the proportion of correctly identified absent connections (also known as the true negative rate; TNR), i.e., the number of true negatives (TN) divided by either the number of non-present connections (*N*) or the summation of true negatives and false positives (FP):

2 $${\text{Specificity }}\left( {{\text{TNR}}} \right)={\text{TN/}}N={\text{TN/}}\left( {{\text{TN}}+{\text{FP}}} \right).$$

Receiver operating characteristic (ROC) curves were created by plotting the sensitivity (TPR) against the specificity (false positive rate; FPR).

(3) The Jaccard index. Because brain networks have a sparse density, sensitivity and specificity are not equally weighted. False positives are more abundant than false negatives, which is not directly reflected by sensitivity and specificity. The Jaccard index, which is one of the most commonly used similarity coefficients (Yin and Yasuda 2006), respects the sparse network density of brain networks by not taking into account the number of true negatives, and determines the amount of overlap between the modeled and ground truth network based on the number of available connections in one or both of the networks:

3 $${\text{Jaccard index}}\;\left( J \right)={\text{TP/}}({\text{TP}}+{\text{FP}}+{\text{FN}}).$$

#### Comparison across fiber lengths

To examine the performance of diffusion-based tractography over different distances, we calculated the Euclidean distance for each pair of regions. The Euclidean distance allowed us to determine the distance between all pairs of regions, irrespective of connecting streamlines. First, x-, y- and z-coordinates (in mm) of the center of gravity of all regions were extracted from the rat brain atlas. Subsequently, the three-dimensional Pythagorean theorem was applied to calculate the Euclidean distance between all pairs of regions, e.g., for regions A and B:

4 $${\text{Euclidean}}\;{\text{distance}}=\sqrt{\left( {{{({x_{\text{A}}} - {x_{\text{B}}})}^2}+{{({y_{\text{A}}} - {y_{\text{B}}})}^2}+{{({z_{\text{A}}} - {z_{\text{B}}})}^2}} \right)}.$$

All region pairs were subdivided into distance categories (i.e., < 2, 2–3, 3–4, 4–5, 5–6, 6–7, 7–8, 8–9, 9–10, 10–11, 11–12, 12–13, 13–14, > 14 mm). We determined sensitivity, specificity and the Jaccard index of DT-based, CSD-based and global tractography for each distance category (i.e., as a proxy of fiber length).

#### Group level thresholding of connectivity matrix

To assess the effect of group average-based connectome reconstruction, which may improve signal-to-noise by including consistently found connections and controlling for particularities of individual rat networks, we included connections based on incidence over all rats (*N* = 10) using different thresholds. For example, thresholding at 0.5 means that for a connection to be included in the final connectome reconstruction at least five out of the ten rats should have a particular connection. We varied thresholds between 0.1 (i.e., a connection has to be found in only one rat to be included) and 1 (i.e., a connection has to be found in all rats to be included), and examined the incidence threshold effect on sensitivity, specificity and the Jaccard index.

#### Streamline thresholding of connectivity matrix

To assess the effect of streamline thresholding, we thresholded connections at different streamline numbers, i.e., 2, 5, 10, 15, 20 and 25 streamlines. Regions with a sub-threshold number of linking streamlines were regarded as not connected (i.e., put at zero). We examined the effects of streamline thresholding on sensitivity, specificity and the Jaccard index.

### Statistical analyses

All descriptive statistical analyses and visualizations were performed in *R 3.2* (https://www.r-project.org/), using the packages *ggplot2, plyr, caret, igraph, network, reshape2* and *sna*.

### Data availability

The datasets generated during and/or analyzed during the current study are available from the corresponding author on reasonable request.

## Results

### Tractography maps

**Fig. 2:**
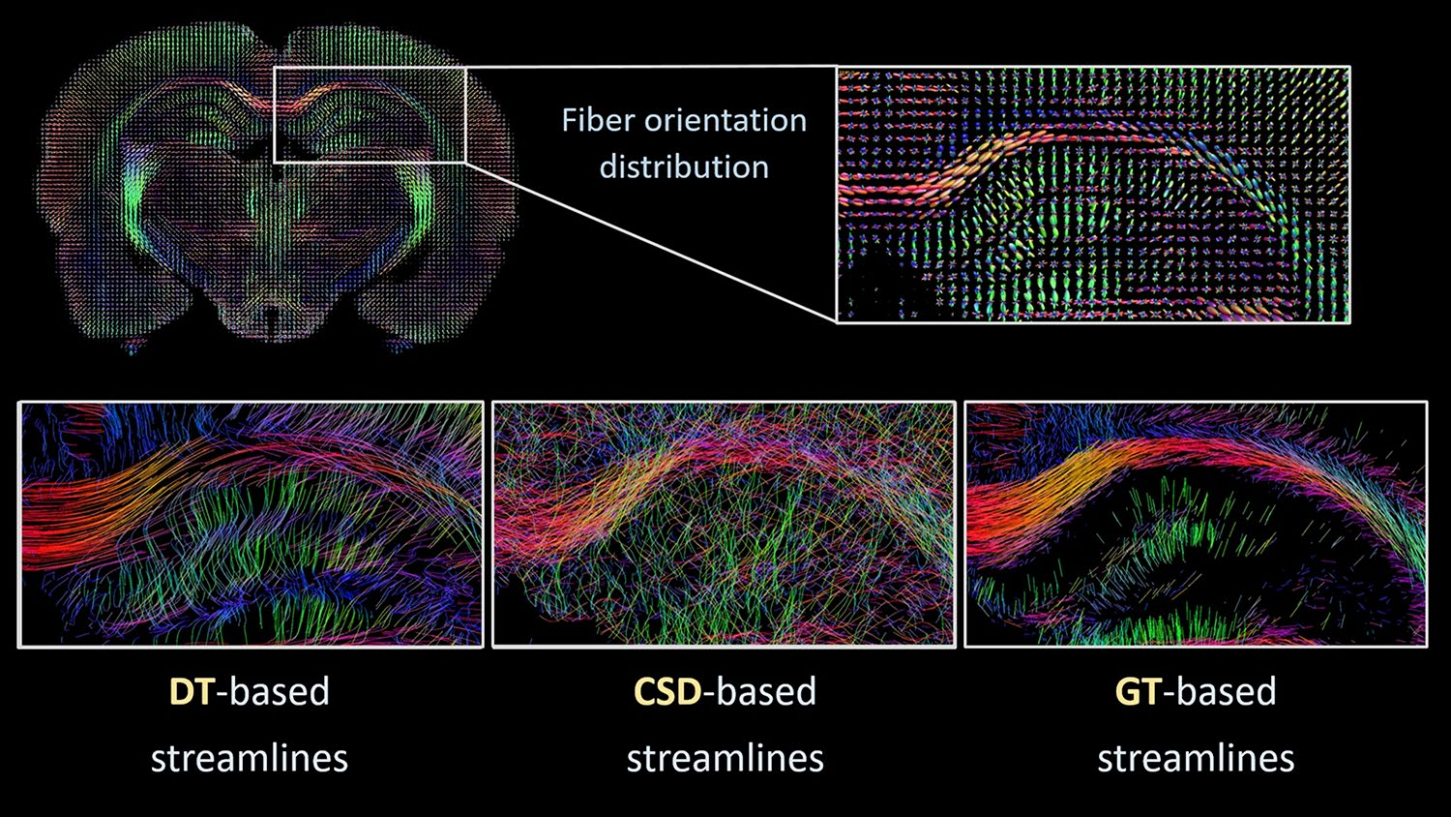
Tractography from high-resolution diffusion MRI of postmortem rat brain. Top: coronal rat brain slice displaying fiber orientation distributions, with an enlarged view of the dorsal hippocampal area. Bottom: representative examples of tract reconstructions in the dorsal hippocampal area, computed with diffusion tensor-based (DT left), constrained spherical deconvolution-based (CSD middle) and global tractography algorithms (GT right)

Figure 2 shows representative examples of fiber reconstructions (streamlines) obtained from the three different tractography algorithms applied to high-resolution rat brain diffusion MRI data. The tractography maps displayed various neuroanatomical pathways with high detail. However, subtle differences in fiber patterns between the three algorithms were apparent. DT-based and global tractography-based streamlines show mostly smoothly delineated fibers, whereas CSD-based streamlines show a more irregular pattern with many crossing fibers. Also, the appearance of reconstructed fibers in different layers of the hippocampal area differs between the three algorithms.

### Accuracy of diffusion tractography-based reconstructions

**Fig. 3:**
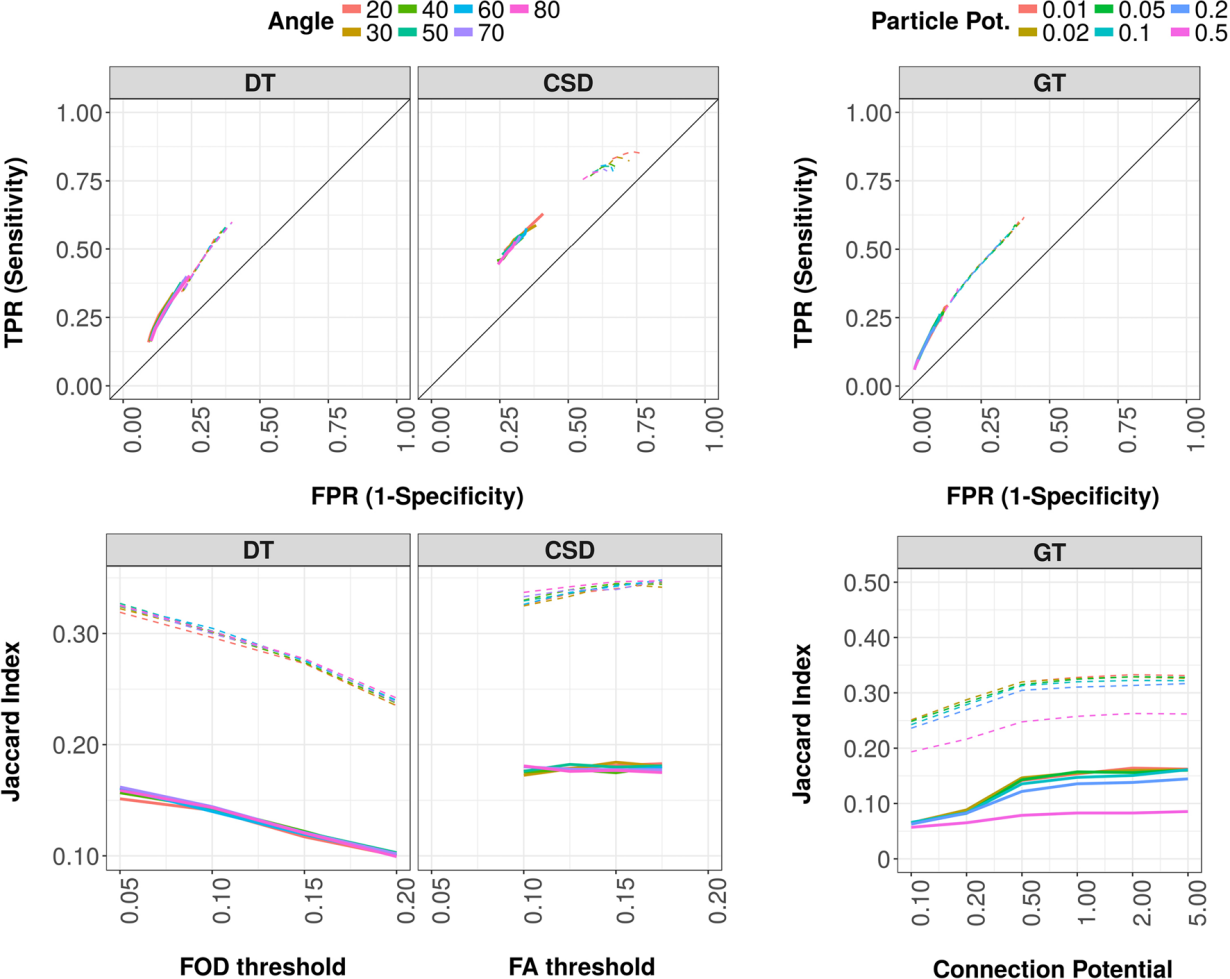
Connectome reconstruction sensitivity, specificity and Jaccard index of DT-based (left), CSD-based (middle) and global tractography (GT) (right). Left and middle graphs: reconstruction sensitivity (true positive rate; TPR) versus 1-specificity (false positive rate; FPR) (top) and Jaccard index (bottom) over FA thresholds (DT-based tractography) and over FOD thresholds (CSD-based tractography), for different angle thresholds (line color) with default step size and 250,000 streamlines. Right graphs: GT-based reconstruction sensitivity versus 1-specificity (top) and Jaccard index (bottom) over connection potentials for different particle potentials (line color). All parameters are plotted for interhemispheric (solid lines) and intrahemispheric (dashed lines) connections separately

To assess the performance of the different diffusion-based tractography algorithms, we validated whole brain connectome reconstructions with neuronal tracer data at the connection level, i.e., by comparing the presence or absence of connections between all possible pairs of regions. This yielded the number of true and false positives as well as true and false negatives, and the resulting sensitivity, specificity and Jaccard index at connectome level. Figure 3 (top) shows reconstruction sensitivity against specificity for the three tractography algorithms. For DT-based tractography, sensitivity against specificity is shown as a function of FA threshold at the default step size (i.e., 15 µm), and for CSD-based tractography as a function of FOD threshold at the default step size (i.e., 75 µm). For global tractography, sensitivity against specificity is shown as a function of connection potential. Figure 3 (bottom) shows the Jaccard index as a function of FA threshold (for DT-based tractography), FOD threshold (for CSD-based tractography) and connection potential (for global tractography). CSD-based tractography led to the highest sensitivity for both inter- and intrahemispheric connections, but the lowest specificity, whereas global tractography resulted in the lowest sensitivity for both inter- and intrahemispheric connections, but the highest specificity. In general, the Jaccard index was higher for both CSD-based and global tractography (specifically at higher connection potentials), but lower for DT-based tractography.

#### DT-based tractography

Figure 3 (left) shows reconstruction sensitivity against specificity of DT-based tractography as a function of FA threshold for different angle thresholds, as a representative example. At connectome level and over all sets of parameter settings, sensitivity varied from 0.22 ± 0.04 to 0.61 ± 0.02, and specificity from 0.60 ± 0.02 to 0.88 ± 0.02 for intrahemispheric connections [mean ± SD (at group level)]. For interhemispheric connections, sensitivity ranged from 0.09 ± 0.02 to 0.42 ± 0.04, and specificity from 0.75 ± 0.02 to 0.96 ± 0.01. Over this range, the network density varied from 0.07 to 0.39. Increase in network density was associated with increase in sensitivity (more true positives), as well as with decrease in specificity, due to more false positives (data not shown).

The FA threshold affected tract reconstruction sensitivity as well as specificity. Increasing FA thresholds (i.e., more strict fiber tracking), e.g., at an angle threshold of 40° and default step size (15 µm), decreased sensitivity from 0.58 to 0.35 for intrahemispheric and from 0.36 to 0.16 for interhemispheric connections, whereas specificity increased from 0.63 to 0.80 for intrahemispheric and from 0.80 to 0.90 for interhemispheric connections. Similarity (i.e., overlap) decreased as indicated by the Jaccard index from 0.32 to 0.24 and from 0.16 to 0.10 for, respectively, intra- and interhemispheric connections. Over all sets of parameter settings, an increase in number of streamlines led to higher sensitivity and lower specificity values. For example, at an FA threshold of 0.15, step size of 15 µm and angle threshold of 50°, from 25,000 to 250,000 streamlines, intrahemispheric sensitivity increased from 0.29 to 0.44, whereas specificity decreased from 0.85 to 0.72. At the same time, the Jaccard index increased with increasing number of streamlines from 0.22 to 0.28. Similar patterns were seen for interhemispheric connections (data not shown). This increase of the Jaccard index is again explained by a considerable increase in true positives (TP) and a decrease in false negatives (FN) (data not shown). The angle threshold and step size had minimal influence on reconstruction sensitivity and specificity. There was a pattern of increasing sensitivity and decreasing specificity at increasing angle thresholds for the lowest FA threshold.

#### CSD-based tractography

Figure 3 (middle) shows reconstruction sensitivity against specificity of CSD-based tractography as a function of FOD threshold for different angle thresholds, as a representative example. At connectome level and over all sets of parameter settings, sensitivity varied from 0.44 ± 0.04 to 0.86 ± 0.02, and specificity from 0.23 ± 0.02 to 0.78 ± 0.02 for intrahemispheric connections [mean ± SD (at group level)]. For interhemispheric connections, sensitivity ranged from 0.15 ± 0.02 to 0.63 ± 0.05, and specificity from 0.60 ± 0.05 to 0.95 ± 0.01. The network density ranged from 0.13 to 0.68 over all network reconstructions.

The FOD threshold had minimal effect on tract reconstruction sensitivity and specificity. However, sensitivity and specificity varied to some extent over different angle thresholds, with a generally declining pattern for sensitivity and an increasing pattern for specificity at increasing angle thresholds. With increasing angle thresholds (i.e., from 20 to 80°), at an FOD threshold of 0.1, interhemispheric sensitivity decreased from 0.63 to 0.53, while specificity increased from 0.59 to 0.69. Intrahemispheric sensitivity decreased from 0.85 to 0.78, whereas specificity increased from 0.24 to 0.37. Over the same range, the Jaccard index slightly decreased from 0.18 to 0.17 for interhemispheric connections, while it slightly increased from 0.32 to 0.34 for intrahemispheric connections. However, there were more false positives and true positives at an angle of 80° for both intra- and interhemispheric connections (data not shown). Over all sets of parameter settings, we measured slightly higher sensitivity, lower specificity and higher Jaccard index values with increasing number of streamlines, explained by an increase in true positives and a decrease in false negatives.

#### Global tractography

Figure 3 (right) shows reconstruction sensitivity against specificity of global tractography as a function of connection potential for different particle potentials. At connectome level and over all sets of parameter settings, sensitivity varied from 0.24 ± 0.02 to 0.62 ± 0.05, and specificity from 0.59 ± 0.03 to 0.90 ± 0.01 for intrahemispheric connections [mean ± SD (at group level)]. For interhemispheric connections, sensitivity ranged from 0.06 ± 0.01 to 0.29 ± 0.03, and specificity from 0.87 ± 0.02 to 0.99 ± 0.01. The density of global tractography-based networks ranged from 0.07 to 0.34.

Sensitivity and specificity were mostly affected by the connection potential. At a particle potential of 0.01, sensitivity increased from 0.07 to 0.29 (interhemispheric) and from 0.33 to 0.62 (intrahemispheric), while specificity decreased from 0.99 to 0.87 (interhemispheric) and from 0.84 to 0.59 (intrahemispheric) with increasing connection potentials (i.e., more connections being formed). The Jaccard index increased from 0.07 to 0.16 for interhemispheric connections and from 0.25 to 0.33 for intrahemispheric connections. Again, the number of true positives increased together with the number of false positives, although there were generally more false positives (data not shown). Particle potential (i.e., amount and distribution of particles) showed less influence on sensitivity and specificity, although for higher particle potentials, (i.e., 0.2 and 0.5) sensitivity was lower and specificity tended to be higher.

### Effect of SIFT on connectome reconstruction

**Fig. 4:**
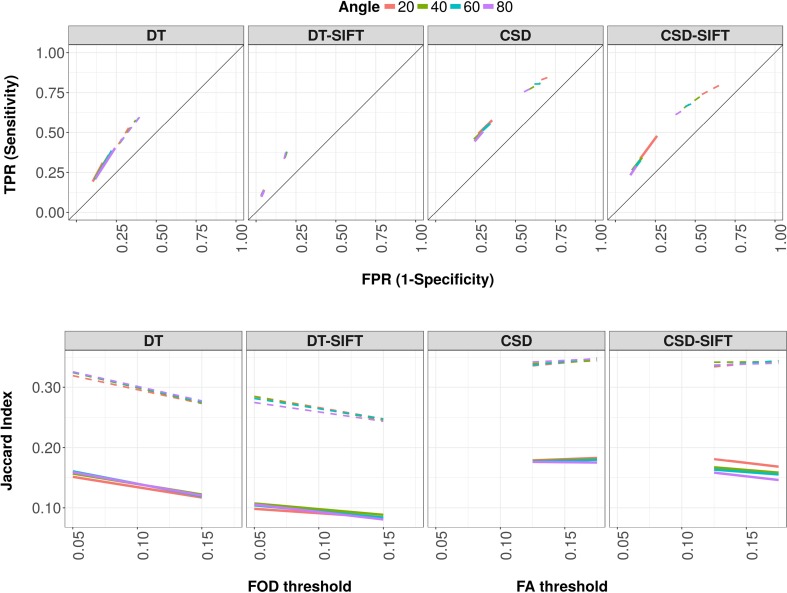
Connectome reconstruction sensitivity, specificity and Jaccard index of DT-based and CSD-based tractography, with and without SIFT correction. Reconstruction sensitivity (true positive rate; TPR) versus 1-specificity (false positive rate; FPR) (top) and Jaccard index (bottom) over FA thresholds (DT and DT-SIFT) and over FOD thresholds (CSD and CSD-SIFT) for different angle thresholds (line color) with default step size and 250,000 streamlines. All parameters are plotted for interhemispheric (solid lines) and intrahemispheric (dashed lines) connections separately

Figure 4 shows sensitivity against specificity, and the Jaccard index over FA thresholds (DT-based tractography) and FOD thresholds (CSD-based tractography) for four different angle thresholds. For both DT- and CSD-based tractography, sensitivity decreased, whereas specificity increased after SIFT. The Jaccard index decreased after applying SIFT. For CSD-based tractography, at an angle threshold of 40°, the interhemispheric sensitivity ranged from 0.42 to 0.54 and the specificity from 0.68 to 0.78, whereas for CSD-SIFT-based tractograms, sensitivity ranged from 0.26 to 0.34 and specificity from 0.83 to 0.90. Similar patterns were found for intrahemispheric connections as well as for DT-based and DT-SIFT-based tractograms.

### Inter- vs. intrahemispheric connections

We determined reconstruction sensitivity and specificity separately for intra- and interhemispheric connections. For all three algorithms, and over all parameter settings, the sensitivity of detecting intrahemispheric connection detection was consistently higher than the sensitivity of interhemispheric connection detection. In contrast, specificity was lower for detection of intrahemispheric connections than for interhemispheric connections. The Jaccard index for intrahemispheric connections was consistently higher as compared to its value for interhemispheric connections.

### Effect of fiber length

**Fig. 5:**
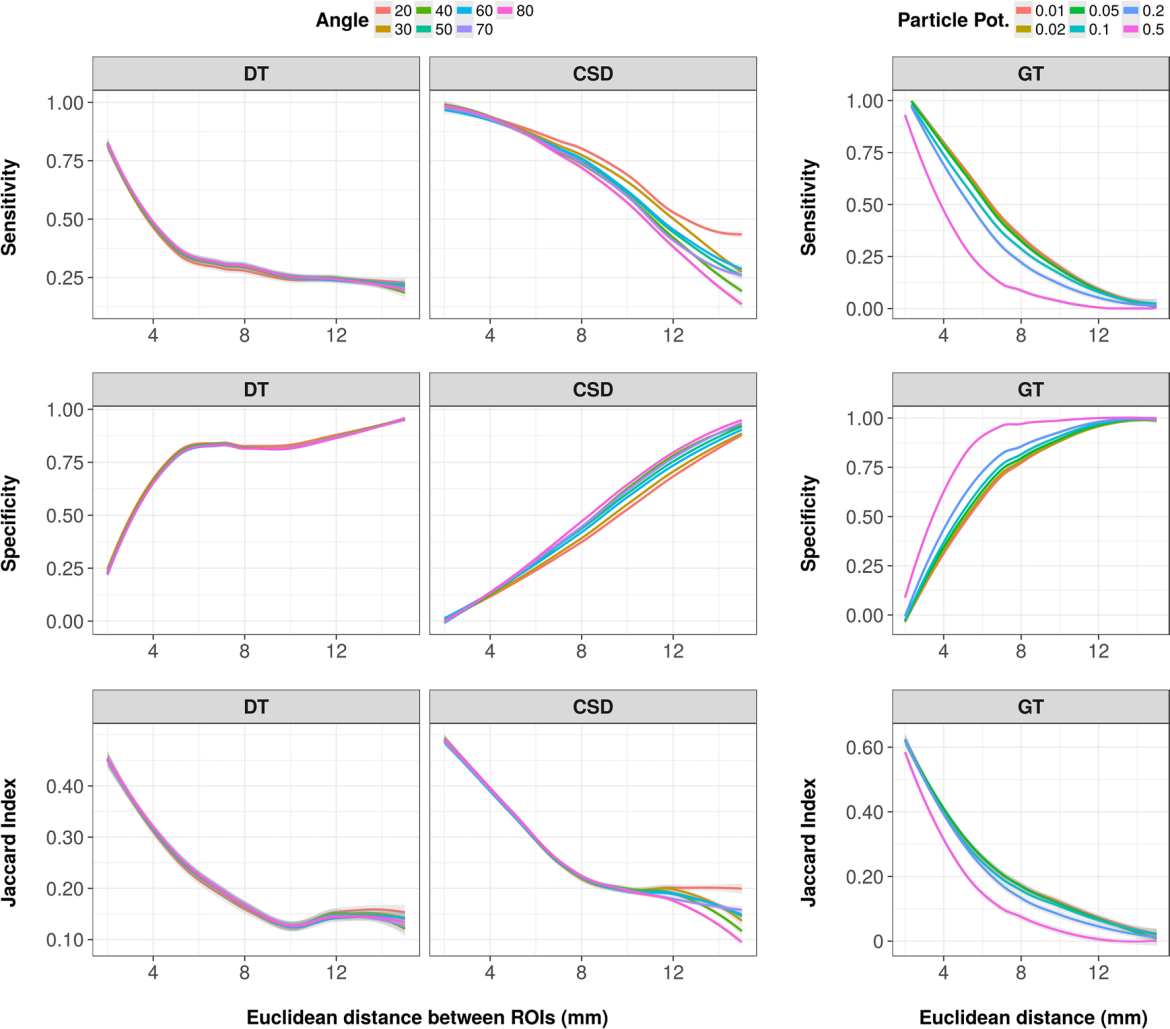
Reconstruction sensitivity, specificity and Jaccard index of DT-based (left), CSD-based (middle) and global tractography (GT) (right). Sensitivity (top), specificity (middle) and Jaccard index (bottom) over Euclidean distance (mm) for DT-based (step size = 15 µm, FA threshold = 0.15) and CSD-based tractography (step size = 75 µm, FOD threshold = 0.125) with 250,000 streamlines and different angle thresholds (line color), and for GT (connection potential = 1) with different particle potentials (line color)

We divided all pairs of connected regions in distance categories for which we determined the tract reconstruction sensitivity, specificity and Jaccard index. Figure 5 shows the average sensitivity, specificity and Jaccard index for DT-based and CSD-based tractography (over all angles at 250,000 streamlines and default step sizes) as well as for global tractography (over all particle potentials and at a connection potential of 1). Overall, with increasing fiber length, there was a non-linear decrease in sensitivity, be it with different patterns for DT-based, CSD-based and global tractography, together with a non-linear increase in specificity. Also, the Jaccard index showed a non-linear decrease with increasing inter-regional distance. Noteworthy was the relatively low sensitivity (< 0.10) and Jaccard index (< 0.05) of global tractography for tracking long-distance fibers. Long-distance streamlines were even lacking in some sets of parameter settings (e.g., at a connection potential of 0.1) for global tractography.

### Effects of group-based analysis

**Fig. 6:**
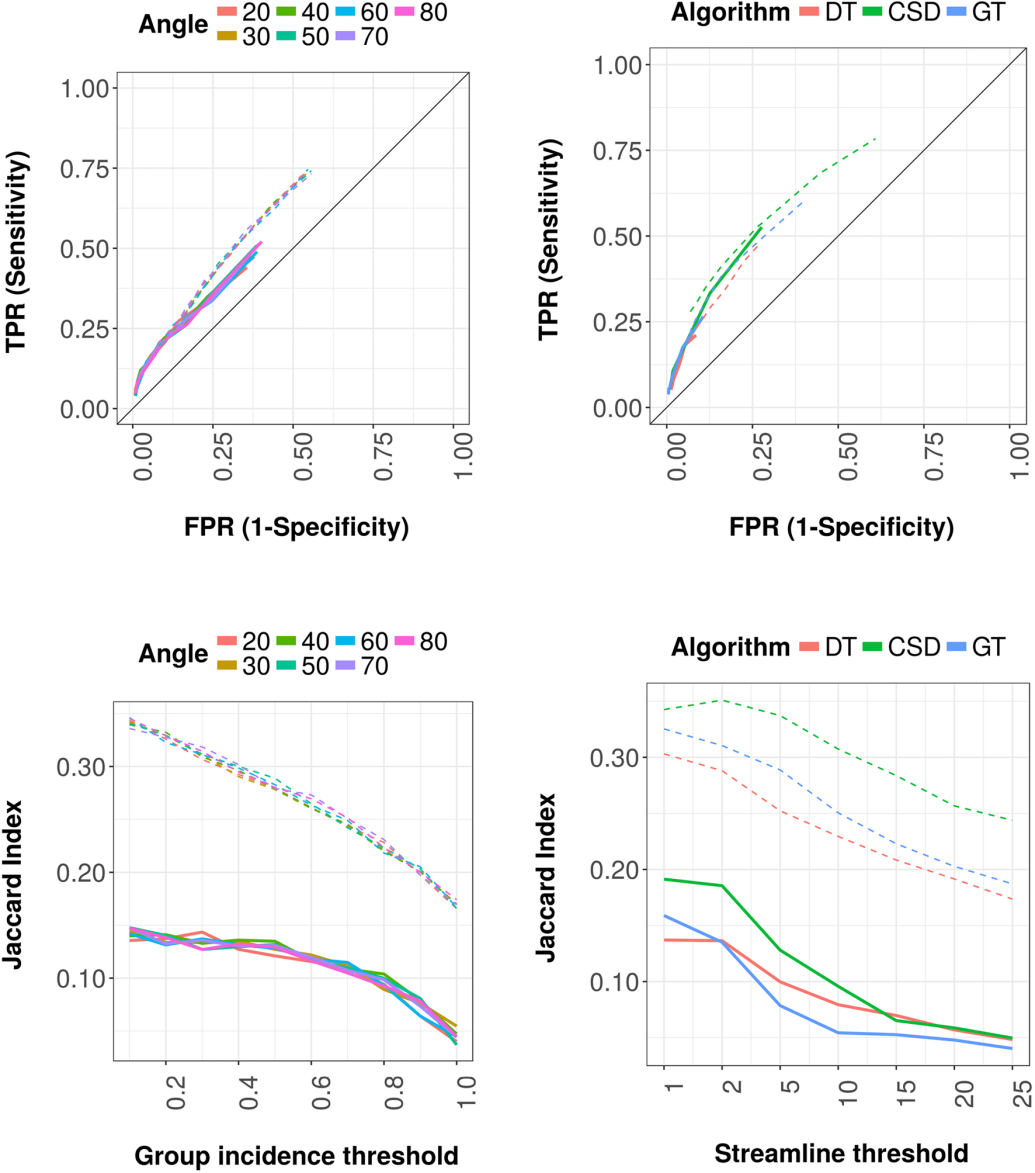
Reconstruction sensitivity, specificity and Jaccard index of DT-based tractography at different group-based incidence thresholds and streamline thresholds. Left graph: reconstruction sensitivity (true positive rate; TPR) versus 1-specificity (false positive rate; FPR) (top) and Jaccard index (bottom) over group incidence thresholds with different angle thresholds (line color) for DT-based tractography (step size = 15 µm, FA threshold = 0.15 and 250,000 streamlines) (left graphs). Right graph: Reconstruction sensitivity (true positive rate; TPR) versus 1-specificity (false positive rate; FPR) (top) and Jaccard index (bottom) over streamline thresholds for DT-based tractography (red; step size = 15 µm, FA threshold = 0.15), CSD (green; step size = 75 µm, FOD threshold = 0.125), with an angle threshold of 40° and 250,000 streamlines, and for global tractography (GT) (blue; connection potential = 1, particle potential = 0.01). All parameters are plotted for interhemispheric (solid lines) and intrahemispheric (dashed lines) connections separately

To analyze the effects of group incidence thresholds, we thresholded an average network at connection level (i.e., based on the number of rats having that specific connection) for DT-based, CSD-based and global tractography. For all tractography algorithms, we found that with increasing threshold (i.e., including connections with increasing incidence—or consistency—across rats) connectome reconstruction sensitivity decreased. At the same time, specificity increased. Figure 6 (left panel) shows sensitivity against specificity (top), and Jaccard indices (bottom) for DT-based tractography (using 250,000 streamlines and an FA threshold of 0.2). Intrahemispheric sensitivity decreased from 0. 75 to 0.20, while specificity increased from 0.44 to 0.92 and interhemispheric sensitivity decreased from 0.52 to 0.04, whereas specificity increased from 0.60 to 0.99. Similar patterns were found for CSD-based and global tractography (Figures S2–S6). For DT-based tractography, the Jaccard index decreased for both intrahemispheric (from 0.35 to 0.17) and interhemispheric connections (from 0.15 to 0.04). Similarly, the Jaccard index of interhemispheric and intrahemispheric connections based on CSD-based tractography showed decreasing patterns, although there were peaks depending on streamline number and location (e.g., interhemispheric connections at 250,000 streamlines and an FOD threshold of 0.175 led to the highest Jaccard index at an incidence threshold of 0.4) (Figure S5). Also, the Jaccard index of inter- and intrahemispheric connections decreased for global tractography, although different patterns can be seen for different particle potentials (i.e., higher particle potentials led to lower Jaccard indices) (Figure S6).

### Effects of streamline thresholds

To analyze the effects of streamline thresholds, we thresholded networks at connection level (i.e., based on the number of streamlines between two regions) for a specific set of parameter settings for DT-based, CSD-based and global tractography (as representative examples), of which connectivity matrices are shown in Figures S7–S9. These connectivity matrices reveal differences in computed connectivity between tractography methods at the level of individual connections. Sensitivity against specificity over different streamline thresholds (i.e., 1, 2, 5, 10, 15, 20 and 25) are shown in Fig. 6 (top right) for specific sets of parameter settings (as representative examples) for the three different algorithms. For all tractography algorithms, we found that with increasing streamline threshold, connectome reconstruction sensitivity decreased, whereas specificity increased (Figures S10 and S11). Although the amount of false positive connections decreased with increasing streamline threshold, the number of true positives decreased as well, specifically at higher thresholds (Figures S12 and S13). For DT-based tractography, the Jaccard index decreased with increasing streamline thresholds, for both interhemispheric (i.e., from 0.14 to 0.05) and intrahemispheric (i.e., from 0.31 to 0.17) connections (Fig. 6). Similar patterns were found for CSD-based and global tractography (Fig. 6).

## Discussion

In this study, we evaluated the accuracy of connectome reconstruction with three different diffusion-based tractography approaches in rat brain. The connectome reconstructions were compared against neuroanatomical tracer data. Despite our ability to track a large variety of brain-wide neuronal fiber connections, we detected a considerable number of false positive and false negative connectivity reconstructions, which increased with increasing fiber lengths. Despite their ability to solve reconstruction issues with crossing fibers, overall performance (i.e., as expressed by the Jaccard index) of CSD-based and global tractography was comparable to conventional DT-based tractography. CSD-based tractography outperformed global tractography for reconstruction of long-distance connections. However, the different tractography approaches presented considerable differences in connectome reconstruction sensitivity and specificity. While DT and GT led to low sensitivity and high specificity, CSD resulted in high sensitivity together with low specificity.

The considerable amount of false positive and false negative connections generated by diffusion-based tractography in rat brain is in line with related studies in macaque brain (Thomas et al. 2014; Azadbakht et al. 2015; Donahue et al. 2016), and mouse brain (Calabrese et al. 2015; Chen et al. 2015), and similar findings have been reported for the human connectome (Maier-Hein et al. 2016). We identified several factors that influence the sensitivity and specificity of connectome reconstruction. The number of streamlines (for CSD- and DT-based tractography) and connection potential (GT) were positively related to sensitivity. Both factors increased the total number of reconstructed connections, leading to a higher true positive rate. Conversely, FA threshold (for DT-based tractography), particle potential (GT), group-based average network thresholding, streamline thresholding and filtering (SIFT) were negatively related to sensitivity. The step size and angle threshold (for CSD- and DT-based tractography) and FOD threshold (for CSD-based tractography) had almost no effect. Improvements in sensitivity were invariably at the expense of specificity and vice versa.

The FA threshold had significantly more effect on connectome reconstruction sensitivity and specificity than the step size and angle threshold, in line with previous research (Azadbakht et al. 2015; Chen et al. 2015). In fact, an increasing FA threshold negatively influenced sensitivity and the Jaccard index, suggesting that an FA threshold should be omitted for this type of datasets and analyses (Azadbakht et al. 2015; Chen et al. 2015). Apparently, the angle thresholds and step sizes—both important factors related to tract curvature—as applied in our study were within plausible neuroanatomical ranges, thereby not affecting the overall connectome reconstructions. However, the angle threshold might still have affected specific pathway reconstructions in the brain (Thomas et al. 2014). Also, further increase (or decrease) of the step size and angle threshold may affect, on their own, or by interaction, subsequent connectome reconstruction.

Interestingly, contrary to the FA threshold (for DT-based tractography), the FOD threshold (for CSD-based tractography) appeared to have no impact on connectome reconstruction sensitivity and specificity. This may be explained by the different natures of DT-based tractography (i.e., deterministic) and CSD-based tractography (i.e., probabilistic) or by the different natures of the FA and FOD thresholds. The FA threshold terminates streamlines in voxels with sub-threshold FA values (i.e., at voxel level), whereas the FOD threshold only prohibits streamline sampling from sub-threshold FOD amplitude peaks within a voxel (i.e., at sub-voxel level), while sampling from other (criteria fulfilling) FOD peaks is still possible. Nevertheless, we expected that an increase in FOD threshold would lead to more rigid delineation of tracts, since streamlines will only follow directions with higher FOD amplitudes (Tournier et al. 2012). However, inspection of our data revealed that many FODs, representing (multiple) crossing fibers, contained multiple high FOD amplitudes. Therefore, to influence the connectome reconstruction sensitivity and specificity, we should apparently increase the FOD thresholds to much higher values (i.e., far beyond the default 0.1 threshold), but this would probably lead to undesirable exclusion of many (truly) crossing fibers. Also, it should be noted that the effects of FOD threshold adjustments may be different across species and datasets.

SIFT filtering has been developed to improve the biological plausibility of final structural connectome reconstructions (Smith et al. 2013, 2015a). However, our study did not reveal an improvement in correspondence between reconstructed connectomes and neuroanatomical connectivity after SIFT correction. As such, our overall results confirm previously reported trade-offs between sensitivity and specificity of tract reconstructions across tractography methods (Bastiani et al. 2012; Thomas et al. 2014; Chen et al. 2015; Donahue et al. 2016). However, we found, as indicated by differing Jaccard indices that the trade-off between reconstruction sensitivity and specificity varies considerably over a range of settings and streamline- or group-based thresholds. This means that a change in the number of false positives through adjustment of tractography parameter settings is not linearly scaled with the change in number of false negatives (and true positives).

Our analyses revealed clear differences in reconstruction sensitivity and specificity dependent on the site (running within or between hemispheres) and length of connections. This is similar to what has previously been reported for macaque brain (Thomas et al. 2014), where reconstruction sensitivity and specificity differed between distinct pathways (and sites) in the brain. The Jaccard index generally increased with higher number of streamlines for intra- and interhemispheric tract reconstructions, despite an increase in false positive tract reconstructions. Our tested DT-based (i.e., deterministic) and CSD-based (i.e., probabilistic) tractography algorithms were affected by connection distance, showing a non-linear decrease in sensitivity and Jaccard index, and a non-linear increase in specificity, with increasing connection distance. CSD-based and global tractography yielded highest Jaccard indices for short-distance connections, which decreased for connections of longer distance. In agreement with previous studies (Zalesky and Fornito 2009; Jbabdi et al. 2015), we found a relatively high rate of false negative reconstructions for connected cortical areas located at large distance from each other, especially for global tractography, which may consequently lead to omissions in identification of important inter-modular hub-node connections. Future studies should focus on elucidation of the exact underlying causes of these variations between and within deterministic and probabilistic tractography approaches. Despite the general trade-off between reconstruction sensitivity and specificity, we observed notable differences between tractography algorithms and parameter settings that should be taken into account for application in connectivity studies and subsequent network analyses. For example, if avoidance of false positives and false negatives are equally important, it may be advisable to choose tractography parameter settings that yield the highest Jaccard index (i.e., a high number of streamlines and a low FA threshold). On the other hand, for modular connectomes such as brain networks, certain network metrics, e.g., clustering coefficient, global efficiency and modularity, have been shown to be more strongly affected by false positives (which tend to arise inter-modularly) than by false negatives (which tend to arise intra-modularly) (Drakesmith et al. 2015; Zalesky et al. 2016). Under such circumstances, an approach that overweighs reconstruction specificity over sensitivity (e.g., with global tractography using a low connection potential, or by applying streamline thresholds to increase specificity), thereby minimizing false positives at the expense of uncovering some true connections, may be preferred (van Wijk et al. 2010; Fornito et al. 2013; Zalesky et al. 2016). Zalesky et al. have recommended that graph-based network analyses should be preferably performed on connectomes with at least about twice the number of false negatives compared to false positives (i.e., the 2:1 rule) (Zalesky et al. 2016). Our data show that specific DT and global tractography parameter settings can satisfy this rule. However, this may be more complicated for CSD, which typically generates high density networks and relatively large numbers of false positives. Lastly, reconstruction sensitivity and specificity may be influenced by streamline thresholding or group-based analysis (i.e., incidence thresholds, which might increase robustness against inter-subject variation), which might be employed to decrease the number of false positive reconstructions to satisfy the suggested 2:1 rule (Zalesky et al. 2016).

It is possible that the mismatch between diffusion-based tractography and neuroanatomical tracers in our (and previous) work may be partly due to incompleteness of neuroanatomical tracer data. For example, the multi-synaptic connections, which are not easily detected with neuronal tracing, might be incorrectly identified as false positives. This actually challenges the concept of neuronal tracing as gold standard method for assessment of neuroanatomical connectivity. It is also conceivable that diffusion tractography findings may guide future neuronal tracing assessments of unknown neuronal pathways. We also did not assess connectivity of subcortical regions, which are less easily co-registerable to the rat brain anatomical atlas, which biases our conclusions towards cortical connectomics. Another point is that we only considered streamline endpoints in determining connectivity between two regions of interest, thereby ignoring the three-dimensional structure of anatomical pathways. It may be argued that this is inappropriate from an anatomical viewpoint, however, this has been the strategy in the majority of structural network studies (which typically focus on network topology and not on exact fiber trajectories). Moreover, a recent study in mice showed that the accuracy of diffusion-based tractography is worse when spatially comparing tractography streamlines with tracer-based connections using 3D co-localization analysis (Calabrese et al. 2015). Lastly, the weights of neuronal connections were not considered in our study. While this was recently done to compare measures of connectivity strength between diffusion tractography and tract tracing data from macaque brain (van den Heuvel et al. 2015; Donahue et al. 2016), most neuroanatomical connections are at least to a certain degree bidirectional and contain nested sub-regions with their own connections and corresponding weights. This complicates the assignment of an absolute or relative measure of connection weight and directionality.

In conclusion, our study shows that conventional deterministic DT-based and more advanced probabilistic CSD-based and global tractography approaches reconstruct the whole-brain connectome with comparable accuracy, be it with considerable amounts of false positive and false negative connections, which strongly depends on the algorithm and parameter settings. Because tractography conditions differ between intra- and interhemispheric connections as well as over different connection distances, algorithms should be chosen purposefully and, if possible, combined to enable most reliable reconstruction of connectomes at large-scale network or whole-brain level. For instance, since global tractography has difficulties with resolving long-distance connections, this long-distance connectivity information could be extracted from different adjacency matrices (based on DT-based tractography). The large number of invalid tract reconstructions we observed in our study, in line with a recent study on human tract reconstructions (Maier-Hein et al. 2016), stresses the relatively ill-posed nature of current tractography approaches and the need for methodological progression. Use of alternative streamline filtering techniques such as SIFT2 (Smith et al. 2015b), inclusion of anatomical constraints (Smith et al. 2012; Lemkaddem et al. 2014) or priors (Yendiki et al. 2011; Christiaens et al. 2014, 2015a), or application of Bayesian connectomics (Hinne et al. 2012; Kasenburg et al. 2016) may lead to further improvement of connectome reconstruction accuracy. Furthermore, the increasing availability of neuronal tracer databases may aid in fine-tuning of diffusion-based tractography settings and applications, which will contribute to the progress of the rising field of connectomics.

## Electronic supplementary material

Below is the link to the electronic supplementary material.

Supplementary material 1 (DOCX 1400 KB)
