# Supplementary material for: Diffusion MRI-based cortical connectome reconstruction: dependency on tractography procedures and neuroanatomical characteristics
Source: Brain Struct Funct. 2018 Feb 20;223(5):2269–85. doi: 10.1007/s00429-018-1628-y (PMC5968063; doi:10.1007/s00429-018-1628-y)
Supplement: Supplementary file 1 [file 429_2018_1628_MOESM1_ESM.docx]

Supplementary Figures

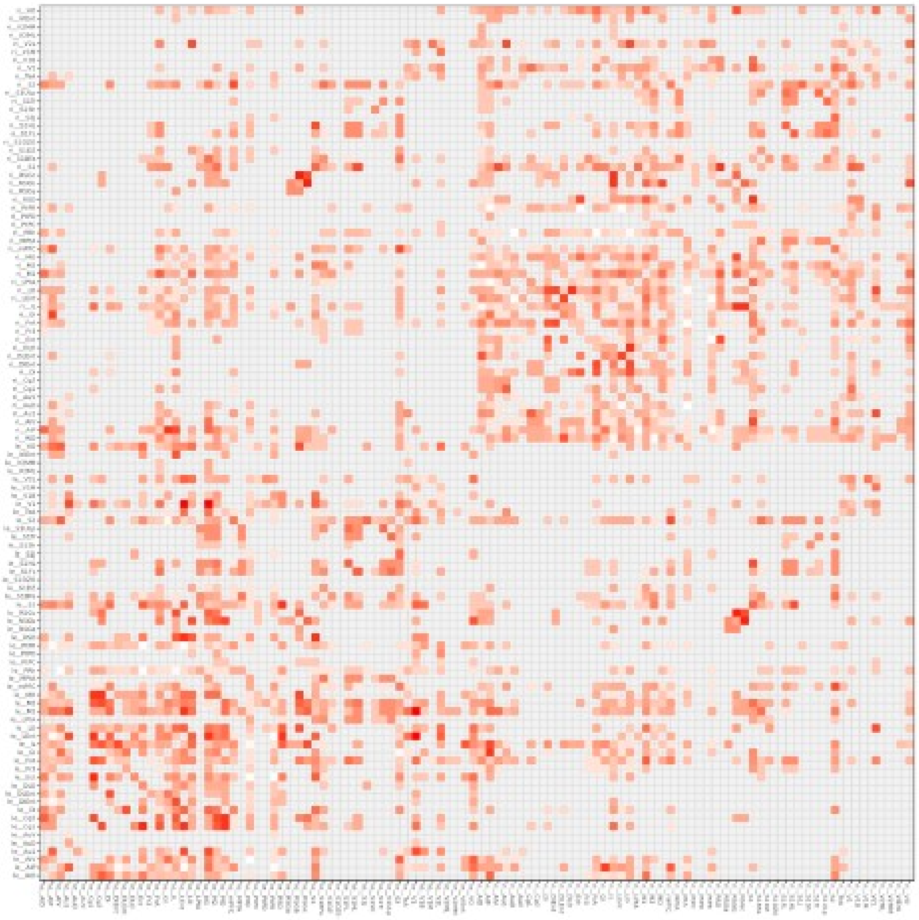

Figure S1. Weighted neuronal tracer-based connectivity matrix. The weighted neuronal tracer-based connectivity matrix (consists of rows and columns representing 106 cortical regions and revealed a network density of 0.27. Connectivity weights are based on the strength of histological connections, ranging from 1 (white) to 8 (red). 'le_[Name]' indicates a node in the left hemisphere, 'ri__[Name]' indicates a node in the right hemisphere.

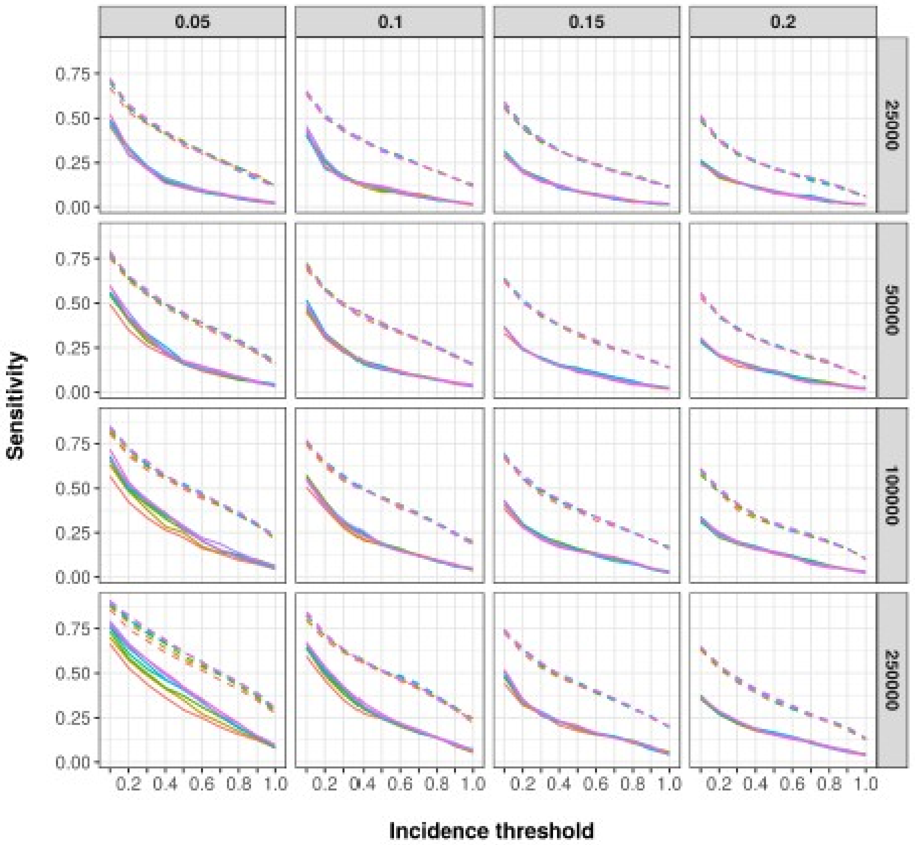

Figure S2. Effect of group-based network thresholding on reconstruction sensitivity of DT-based tractography. Sensitivity is shown for interhemispheric (solid lines) and intrahemispheric (dashed lines) connections. The network proportion threshold (x-axis) reflects the proportion of rats with a specific connection. Sensitivity is shown over different FA thresholds (columns), number of streamlines (rows) and different angle thresholds (line colour).

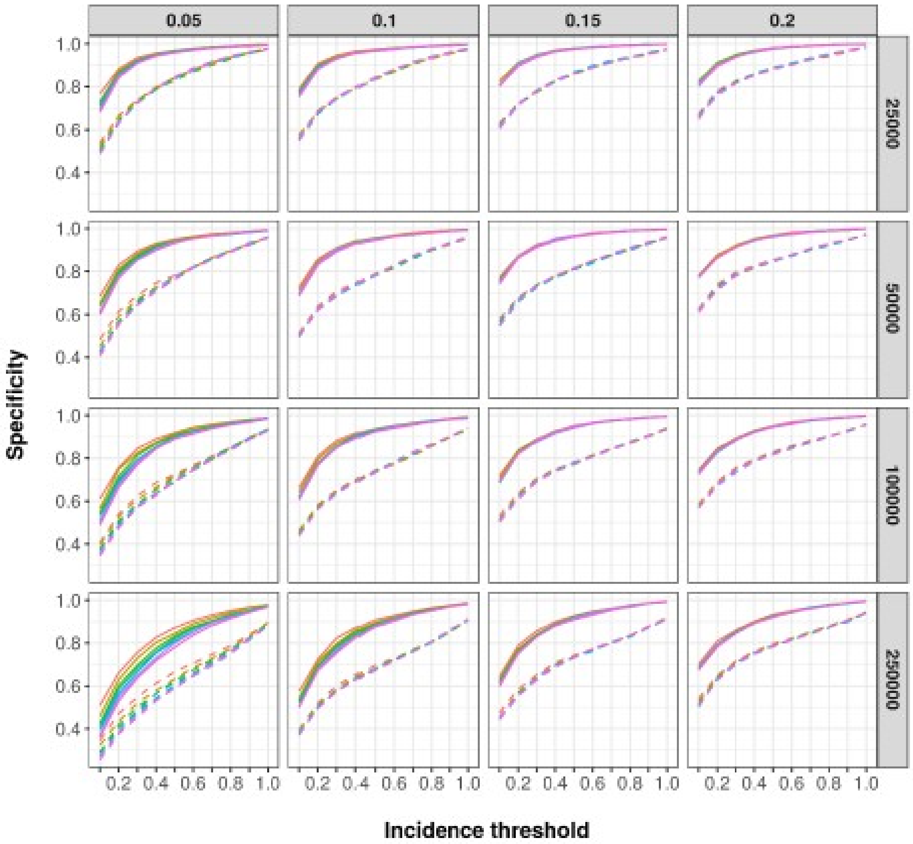

Figure S3. Effect of group-based network thresholding on reconstruction specificity of DT-based tractography. Specificity is shown for interhemispheric (solid lines) and intrahemispheric (dashed lines) connections. The network proportion threshold (x-axis) reflects the proportion of rats with a specific connection. Sensitivity is shown over different FA thresholds (columns), number of streamlines (rows) and different angle thresholds (line colour).

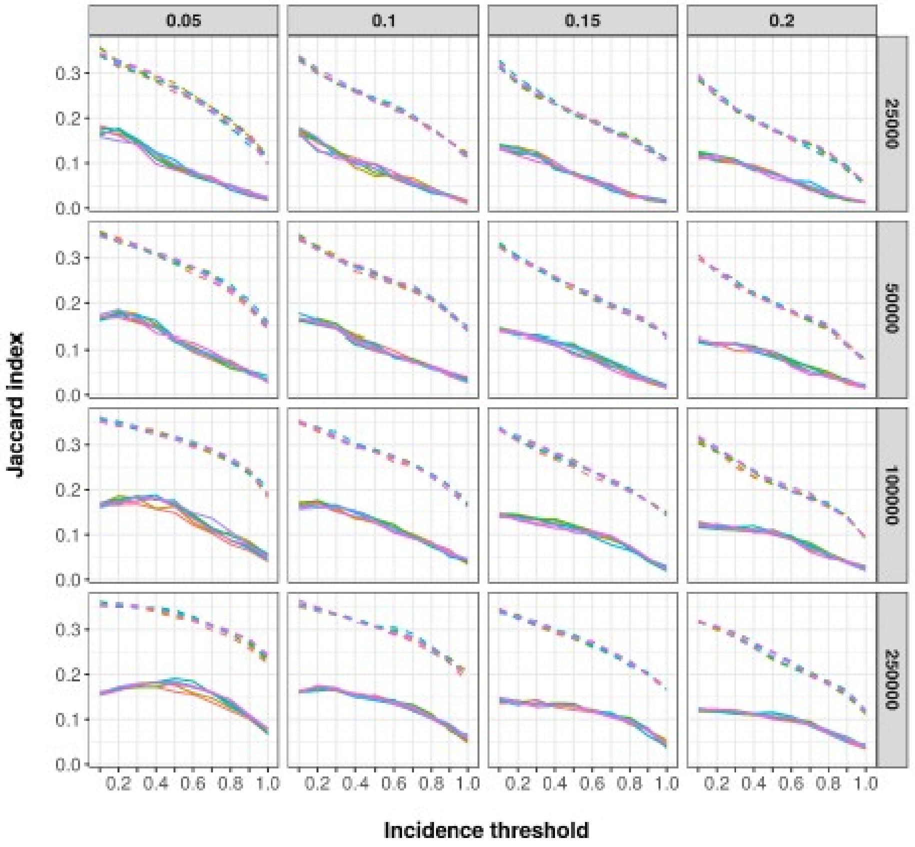

Figure S4. Effect of group-based network thresholding on reconstruction Jaccard index of DT-based tractography. Jaccard index is shown for interhemispheric (solid lines) and intrahemispheric (dashed lines) connections. The network proportion threshold (x-axis) reflects the proportion of rats with a specific connection. Sensitivity is shown over different FA thresholds (columns), number of streamlines (rows) and different angle thresholds (line colour).

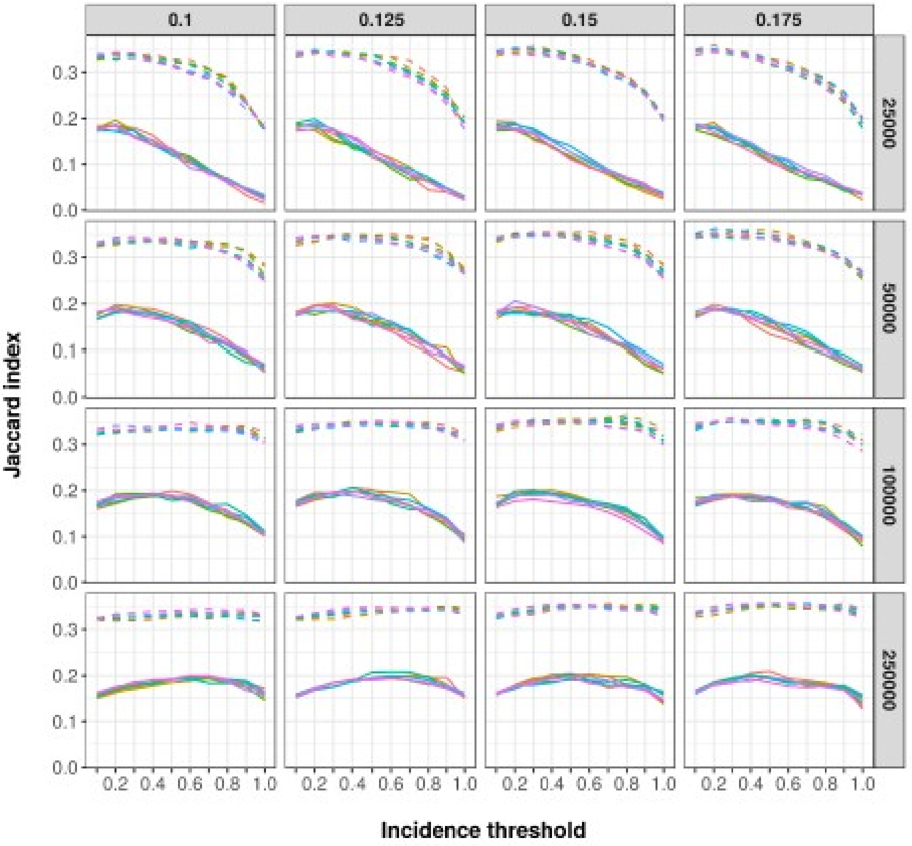

Figure S5. Effect of group-based network thresholding on reconstruction Jaccard index of CSD-based tractography. Jaccard index is shown for interhemispheric (solid lines) and intrahemispheric (dashed lines) connections. The network proportion threshold (x-axis) reflects the proportion of rats with a specific connection. Sensitivity is shown over different FOD thresholds (columns), number of streamlines (rows) and different angle thresholds (line colour).

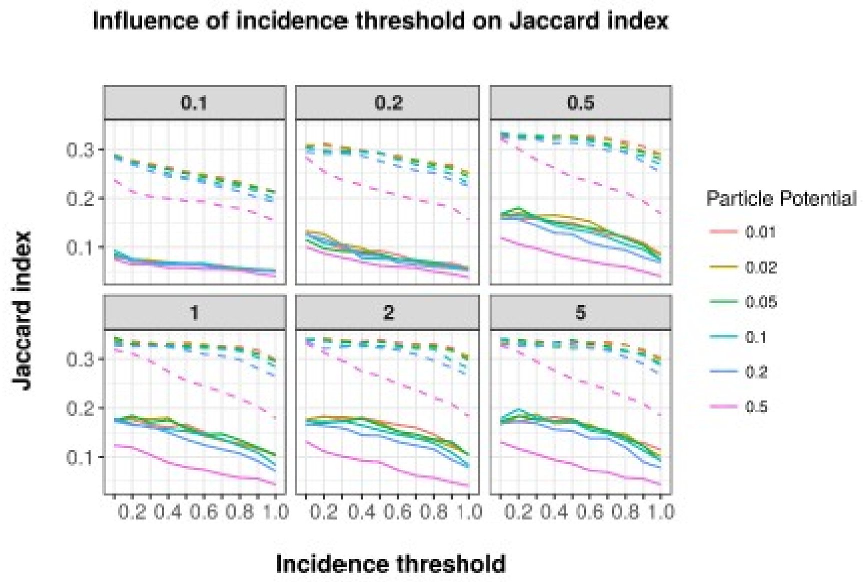

Figure S6. Effect of group-based network thresholding on reconstruction Jaccard index of GT-based tractography. Jaccard index is shown for interhemispheric (solid lines) and intrahemispheric (dashed lines) connections. The network proportion threshold (x-axis) reflects the proportion of rats with a specific connection. Sensitivity is shown over different connection potentials (top) and different particle potentials (line colour).

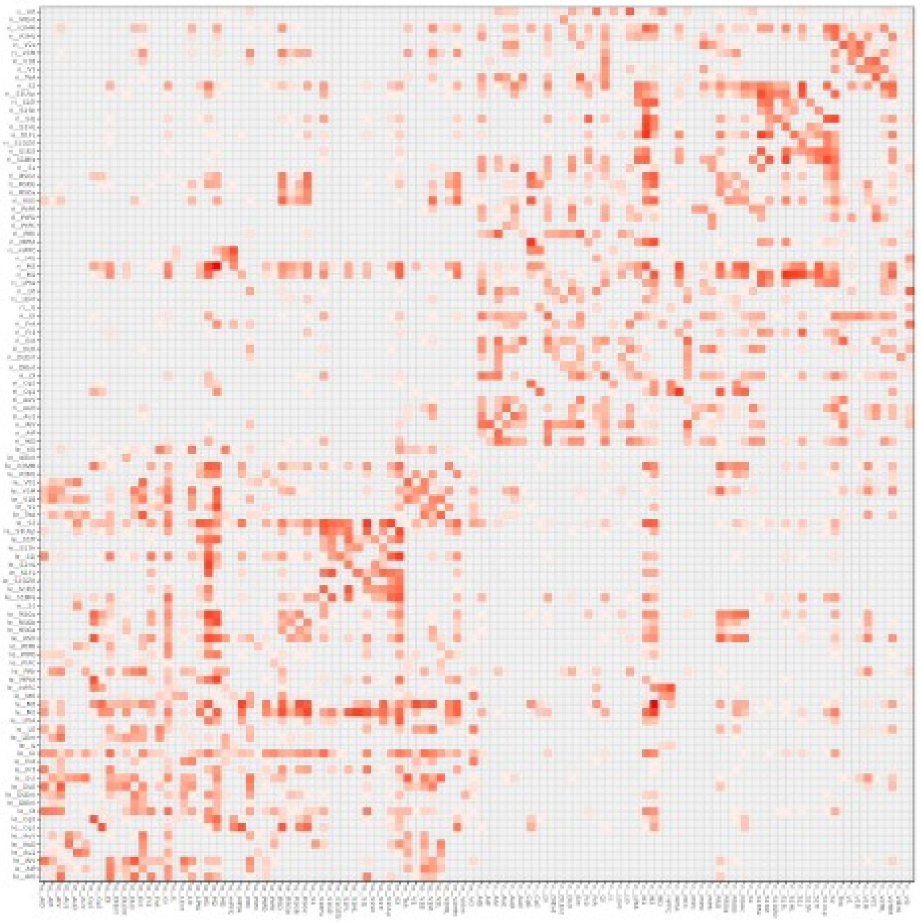

Figure S7. Weighted DT-based connectivity matrix. A structural connectivity matrix in which the rows and columns represent cortical regions (N = 106). Connectivity weights are based on the log-transformed number of streamlines connecting two regions, ranging from transparent (no streamlines) to dark red (maximum number of streamlines). 'le_[Name]' indicates a node in the left hemisphere, 'ri__[Name]' indicates a node in the right hemisphere. DT parameter settings for this particular matrix (density = 0.22): FA threshold = 0.15, angle threshold = 40°, step-size = 15 μm, number of streamlines = 250,000.

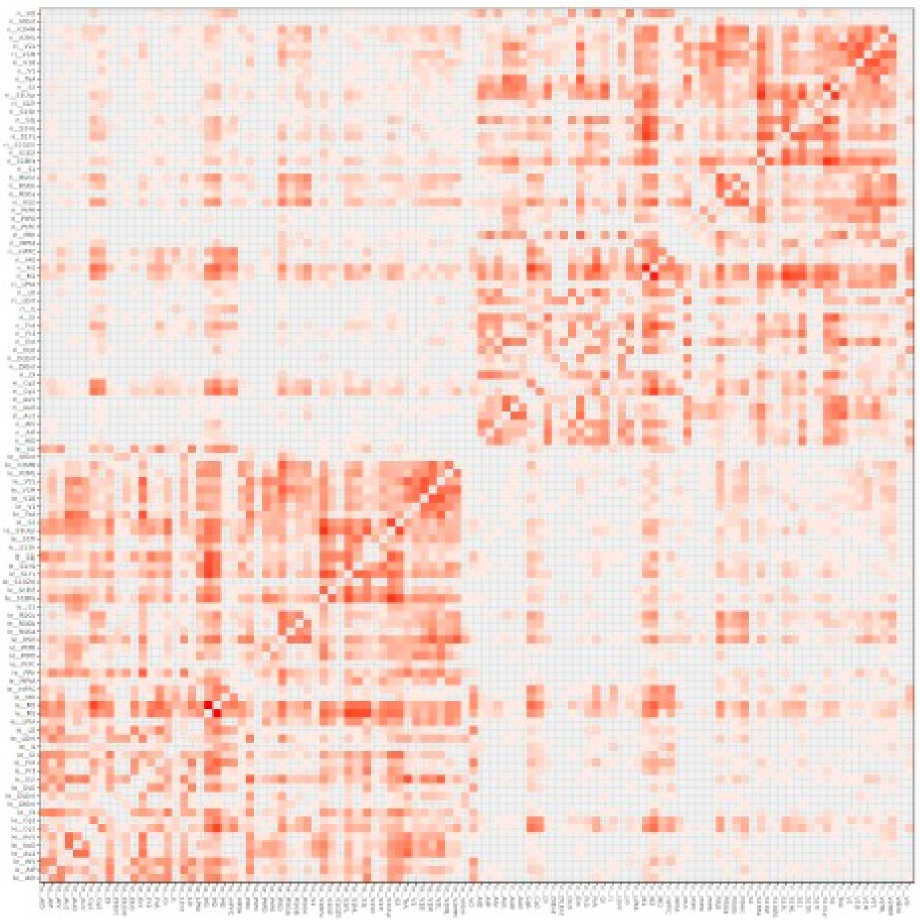

Figure S8. Weighted CSD-based connectivity matrix. A structural connectivity matrix in which the rows and columns represent cortical regions (N = 106). Connectivity weights are based on the log-transformed number of streamlines connecting two regions, ranging from transparent (no streamlines) to dark red (maximum number of streamlines). 'le_[Name]' indicates a node in the left hemisphere, 'ri__[Name]' indicates a node in the right hemisphere. CSD parameter settings for this particular matrix (density = 0.54): FOD threshold = 0.125, angle threshold = 40°, step-size = 75 μm, number of streamlines = 250,000.

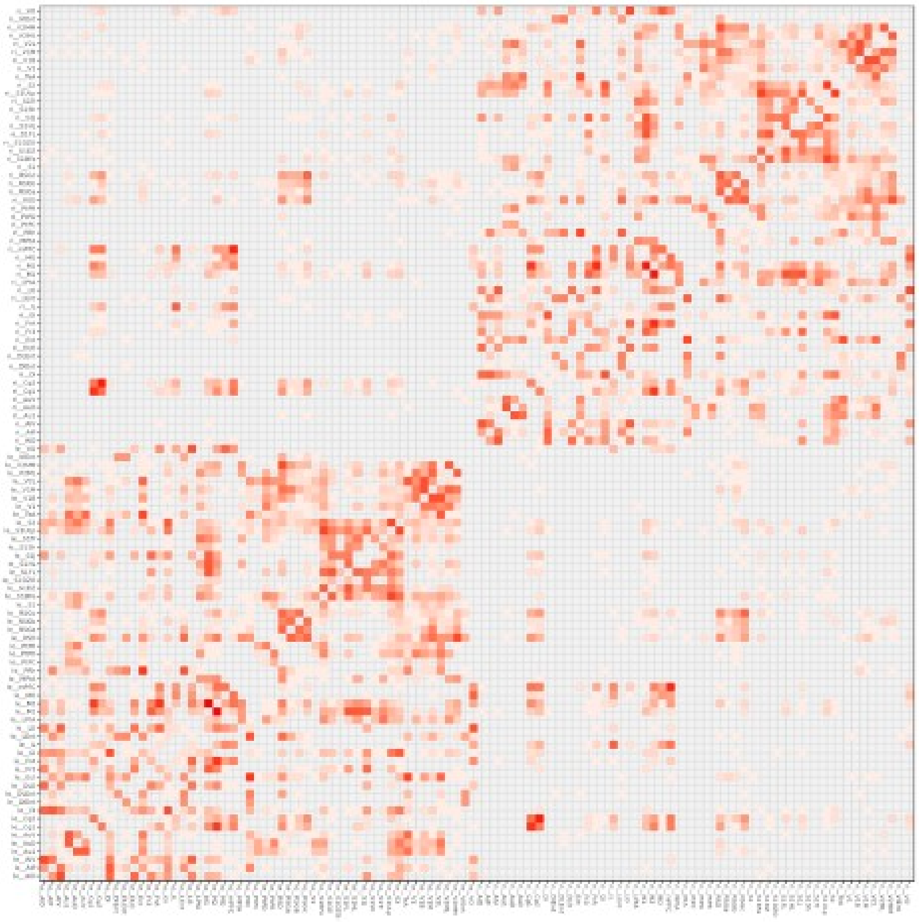

Figure S9. Weighted GT-based connectivity matrix. A structural connectivity matrix in which the rows and columns represent cortical regions (N = 106). Connectivity weights are based on the log-transformed number of streamlines connecting two regions, ranging from transparent (no streamlines) to dark red (maximum number of streamlines). 'le_[Name]' indicates a node in the left hemisphere, 'ri__[Name]' indicates a node in the right hemisphere. GT parameter settings for this particular matrix (density = 0.29): particle potential = 0.01, connection potential = 1.

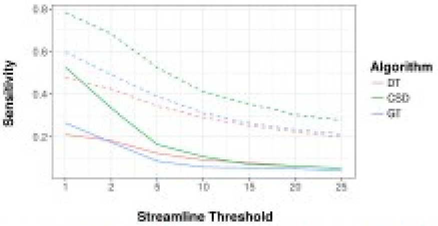

Figure S10. Effect of streamline thresholding on reconstruction sensitivity of DT-, CSD- and GT-based tractography. Sensitivity is shown for interhemispheric (solid lines) and intrahemispheric (dashed lines) connections. The streamline threshold reflects the minimum number of streamlines between two regions to be considered as a connection (i.e. sub-threshold streamline connections were excluded). Parameter settings for DT-, CSD- and GT-based tractography can be found in the legends of Figures S7, S8 and S9, respectively.

s

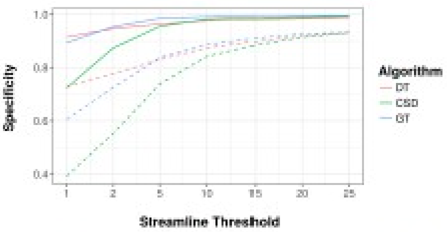

Figure S11. Effect of streamline thresholding on reconstruction specificity of DT-, CSD- and GT-based tractography. Specificity is shown for interhemispheric (solid lines) and intrahemispheric (dashed lines) connections. The streamline threshold reflects the minimum number of streamlines between two regions to be considered as a connection (i.e. sub-threshold streamline connections were excluded). Parameter settings for DT-, CSD- and GT-based tractography can be found in the legends of Figures S7, S8 and S9, respectively.

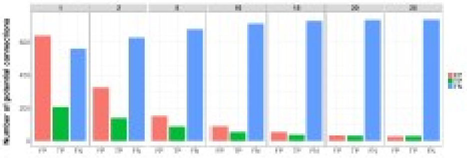

Figure S12. Influence of streamline-thresholds on interhemispheric DT-based tractography reconstruction accuracy. Different streamline thresholds (top) have different effects on the reconstruction accuracy reflected by the number of false positives (FP), true positives (TP) and false negatives (FN) shown at bottom.

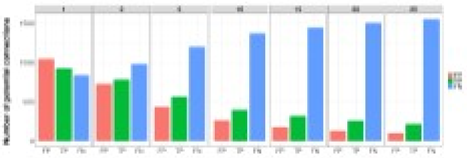

Figure S13. Influence of streamline-thresholds on intrahemispheric DT-based tractography reconstruction accuracy. Streamline threshold (top) affect the accuracy reflected by the number of false positives (FP), true positives (TP) and false negatives (FN) shown at.
